# MemCLR-GANomaly: An Unsupervised Anomaly Detection Approach for Resin Cut-Off Wheel Surfaces

**DOI:** 10.3390/s26196148

**Published:** 2026-09-28

**Authors:** Xiaona Song, Runqing Zhang, Kaixuan Lv, Lijun Wang, Jing Liu, Ying Zhu

**Affiliations:** 1 School of Mechanical Engineering, North China University of Water Resources and Electric Power, Zhengzhou 450046, China; songxiaona1@126.com (X.S.); lv20031218@126.com (K.L.); wanglijun@ncwu.edu.cn (L.W.); liuj1998106@163.com (J.L.); 2 China Railway Engineering Equipment Group Co., Ltd., Zhengzhou 450016, China; zyx116@163.com

**Keywords:** resin cut-off wheel surface inspection, unsupervised anomaly detection, SimCLR, dynamic memory bank, adaptive fusion network

## Abstract

Unsupervised anomaly detection is critical for industrial surface inspection, especially for resin cut-off wheels where defect samples are scarce and morphologically diverse. GANomaly serves as a classic reconstruction-based baseline using latent vector discrepancy, yet its single-score mechanism conflates pixel-level structural, feature-level semantic, and distribution-level statistical anomalies into one scalar, causing inherent detection blind spots for complex industrial defects. To address this issue, we propose a three-dimensional anomaly scoring framework instantiated as MemCLR-GANomaly. Specifically, a multi-scale region error module captures pixel-level structural anomalies; a SimCLR-enhanced contrastive discrepancy branch quantifies feature-level semantic deviations; and a dynamic memory bank models distribution-level statistical normality. An adaptive fusion network integrates the three indicators with sample-dependent weights to generate the final anomaly score. Experiments on the self-built Resin Cut-off Wheel Dataset achieve image- and pixel-level AUROC values of 0.995 and 0.988, with a false positive rate as low as 0.012. On a custom partition of the public MVTec AD dataset, the method attains an image-level AUROC of 0.948 and a pixel-level AUROC of 0.936. With an inference speed of 42.3 FPS and a computational cost of 3.9 GFLOPs, the proposed method meets the real-time requirements of online industrial inspection for resin cut-off wheel production lines.

## 1. Introduction

Grinding is a core finishing process in precision manufacturing, and reinforced resin cut-off wheels are widely used in high-speed metal cutting for their high efficiency and low cost. Their multilayer composite structure leads to diverse manufacturing defects such as abrasive contamination, mesh misalignment and delamination, which degrade machining quality and may cause catastrophic failure at high speeds. Therefore, reliable surface defect inspection is essential for industrial quality control [1,2].

Traditional resin cut-off wheel inspection relies on manual visual check or contact monitoring techniques like acoustic emission, which suffer from low efficiency, subjective judgment and poor scalability in automated production [3,4]. More broadly, machine-learning-based sensor monitoring converts acquired signals into task-specific decisions, including condition assessment, damage detection and localization [5]. Although deep learning has greatly advanced surface inspection, supervised methods require massive annotated defect samples. Given the extremely low defect rate and diverse defect morphologies in real production, unsupervised visual anomaly detection trained solely on normal samples has become a promising solution [6,7,8].

Existing unsupervised methods mainly fall into three paradigms. Reconstruction-based approaches such as GANomaly and DRAEM identify anomalies via reconstruction inconsistency, but powerful generators tend to inpaint abnormal regions, causing missed detection of subtle defects [9,10]. Feature distribution modeling methods including PaDiM and PatchCore achieve competitive performance via pre-trained representations, yet their robustness degrades under domain shifts caused by batch production fluctuations [11,12]. Memory-augmented methods store normal prototypes for similarity matching, but most adopt static update strategies that cannot adapt to evolving production distributions [13,14]. In addition, contrastive learning has been widely adopted to enhance feature discriminability [15,16].

Nevertheless, most existing methods rely on a single dominant representation space, leading to incomplete characterization of complex industrial anomalies. For resin cut-off wheels, defects simultaneously exhibit pixel-level texture distortions, feature-level semantic inconsistencies and distribution-level quality fluctuations. Compressing such heterogeneous cues into a single scalar score causes inevitable information aliasing, resulting in inherent detection blind spots and elevated false alarm rates in practical deployment.

To address this limitation, we propose a three-dimensional anomaly scoring principle as the theoretical foundation of our framework. We posit that industrial anomalies manifest as composite deviations across three complementary dimensions: the pixel-level structural dimension $D_{p}$ for local geometric distortions, the feature-level semantic dimension $D_{f}$ for appearance inconsistencies, and the distribution-level statistical dimension $D_{d}$ for global domain shifts. This formulation preserves the heterogeneity of anomaly cues throughout the scoring pipeline and effectively alleviates the information aliasing problem of single-score mechanisms.

Guided by this principle, we develop MemCLR-GANomaly, a unified tri-branch unsupervised anomaly detection framework for resin cut-off wheel surface inspection. It integrates a multi-scale region error module, a SimCLR-enhanced contrastive discrepancy branch and a dynamic memory bank with two-stage update strategy, and fuses the three-dimensional scores via a lightweight adaptive fusion network with sample-dependent weights.

The main contributions of this work are summarized as follows:

1. A three-dimensional anomaly scoring principle that explicitly disentangles pixel-level structural, feature-level semantic and distribution-level statistical anomaly cues, fundamentally addressing the information aliasing problem of conventional single-score mechanisms.
2. A complete MemCLR-GANomaly framework integrating multi-scale reconstruction, contrastive learning, dynamic memory bank and adaptive score fusion into a synergistic detection pipeline.
3. Comprehensive experiments on the self-built Resin Cut-off Wheel Dataset demonstrate the effectiveness of the proposed method. Evaluation on a custom partition of MVTec AD provides an additional assessment of its applicability beyond the target dataset. With an inference speed of 42.3 FPS and a computational cost of 3.9 GFLOPs, the method meets the real-time requirements of industrial online inspection.

The remainder of this paper is organized as follows. Section 2 reviews related work on resin cut-off wheel inspection and unsupervised anomaly detection. Section 3 details the three-dimensional scoring principle and the MemCLR-GANomaly framework. Section 4 evaluates the method on the self-built Resin Cut-off Wheel Dataset and the public MVTec AD benchmark. Finally, Section 5 concludes the paper.

## 2. Related Work

This work integrates resin cut-off wheel quality inspection with unsupervised visual anomaly detection. This section reviews the state of the art of resin cut-off wheel surface inspection and mainstream unsupervised anomaly detection paradigms, and clarifies the research gap targeted by this study.

### 2.1. Surface Defect Inspection of Resin Cut-Off Wheels

Resin cut-off wheels are widely used in high-speed cutting and precision manufacturing for high efficiency, low cost and superior cutting performance, whose manufacturing quality directly determines machining accuracy, productivity and operational safety. Extensive research and systematic reviews have addressed grinding wheel wear monitoring and process evaluation via acoustic emission, force, vibration and multi-sensor fusion, supporting in-process degradation tracking [2,3,17]. From a broader monitoring perspective, machine learning connects sensor representations with condition assessment, detection and localization tasks across different application domains [5]. These studies provide a complementary context for visual inspection, whereas in-process wear monitoring does not directly replace surface defect inspection of finished wheels.

Computer vision has become the mainstream industrial quality control solution. Existing wheel vision studies adopt handcrafted texture descriptors or supervised deep learning detectors for defect and wear identification [6,18]. While effective in specific scenarios, they rely heavily on large annotated datasets and lack robustness to varying defect morphologies or production conditions. In practice, resin cut-off wheel defects (e.g., abrasive contamination, mesh damage, delamination, printing flaws, flange deformation) are rare but highly diverse in scale, texture and location, making full annotation prohibitively labor-intensive.

Unsupervised industrial anomaly detection has attracted growing attention, as it trains solely on normal samples. Public benchmarks like MVTec AD and VisA have greatly advanced algorithm development and evaluation [19,20]. However, most studies focus on general industrial products and standard datasets, with scarce research on resin cut-off wheel inspection. Critically, resin cut-off wheels’ complex multi-region structure and diverse defects cause concurrent pixel-level texture variations, feature-level structural changes and distribution-level manufacturing fluctuations, posing severe challenges to existing methods.

### 2.2. Reconstruction-Based Anomaly Detection

Reconstruction-based methods are among the earliest and most widely used unsupervised anomaly detection paradigms, trained on normal samples and identifying anomalies through input–reconstruction discrepancies. GANomaly adopts an encoder–decoder–encoder architecture to detect anomalies through latent representation inconsistency [9]. f-AnoGAN improves inference efficiency through explicit latent mapping, while Skip-GANomaly introduces skip connections to preserve structural details during reconstruction [21,22]. DRAEM combines reconstruction with discriminative embedding to improve localization, and MemAE retrieves normal prototypes from an external memory to constrain reconstruction [10,13]. Related approaches have also been explored in hyperspectral anomaly detection. Frequency-to-spectrum mapping GAN adopts a semisupervised adversarial framework, while the global feature-injected blind-spot network incorporates global information into blind-spot modeling [23,24]. More recently, a nonlocal and local feature-coupled self-supervised network combines complementary contextual information for hyperspectral anomaly detection [25]. These studies illustrate the use of different representations and learning strategies for anomaly detection beyond industrial RGB images.

Despite their effectiveness, these methods have notable limitations in industrial inspection. First, powerful generators tend to inpaint abnormal regions as normal textures, weakening reconstruction residuals and causing missed detection of subtle defects such as slight abrasive contamination, tiny mesh fractures and minor printing imperfections on resin cut-off wheels. Second, most approaches rely on a single anomaly score derived from reconstruction error or latent feature discrepancy, which fails to simultaneously capture pixel-level appearance, feature-level structural and distribution-level manufacturing anomalies. Their performance thus degrades on complex textured industrial products with diverse defects, motivating research on multi-dimensional anomaly representation and adaptive fusion strategies.

### 2.3. Feature Distribution Modeling

Different from reconstruction-based paradigms, feature distribution modeling methods characterize normal feature statistics from pre-trained deep networks and detect anomalies as deviations from the learned distribution. PaDiM models multivariate Gaussian distributions of patch features to realize training-free anomaly localization [11]. PatchCore further boosts detection performance via a memory bank of representative normal features with nearest-neighbor matching inference [12]. For efficiency improvement, CFLOW-AD frames detection as conditional density estimation using normalizing flows, while FastFlow adopts lightweight 2D flows to achieve real-time anomaly localization [26,27]. To further adapt to industrial scenarios with extremely limited normal samples, normalizing flow-based distribution modeling has also been extended to few-shot anomaly detection, maintaining competitive detection performance with significantly reduced data dependency [28]. RD4AD introduces reverse knowledge distillation to reconstruct normal features from one-class embeddings, and EfficientAD reduces computational cost via an efficient student-teacher architecture while retaining competitive accuracy [29,30]. Recent works have further investigated multi-class and universal anomaly detection with compact normal representations or intrinsic normal prototypes [8,31].

Despite state-of-the-art results on benchmark datasets, these methods rely on fixed pre-trained feature distributions, and their robustness degrades under industrial domain shifts and large intra-class variations. For resin cut-off wheels, inherent manufacturing fluctuations in abrasive distribution, resin color and fiber texture create substantial appearance diversity among normal samples. Such variations often overlap with abnormal patterns in feature space, making pure distribution modeling insufficient to distinguish subtle defects from normal production variations.

### 2.4. Memory-Augmented and Contrastive Representation Learning

Memory learning effectively models normal patterns for industrial anomaly detection. MemAE first introduces an external memory module to retrieve normal prototypes for reconstruction, suppressing abnormal region recovery [13]. Memory-guided Normality Learning optimizes prototype selection via adaptive retrieval to boost discriminability of normal representations [14]. PatchCore builds a representative memory bank of normal patch features for nearest-neighbor anomaly detection, achieving strong performance on industrial benchmarks [12]. ReConPatch further integrates memory mechanisms with contrastive learning to improve patch-level representation quality and localization accuracy [16]. While alleviating normal intra-class variations, these methods focus mainly on distribution-level normality modeling, lacking explicit integration of complementary pixel- and semantic-level anomaly cues.

Meanwhile, contrastive learning underpins discriminative visual representation learning. SimCLR promotes feature consistency across augmented views while distinguishing different instances, enabling representation learning without manual annotations [15]. CutPaste learns anomaly-sensitive representations through a self-supervised task that distinguishes normal images from cut-and-paste augmentations [32]. CLIP enables transferable visual learning through large-scale vision–language pre-training and image–text alignment [33]. WinCLIP, PromptAD and AnomalyCLIP further exploit vision–language representations and prompting strategies for zero-/few-shot industrial anomaly detection [34,35,36]. Beyond surface texture anomalies, research has extended to logical structural anomalies involving component arrangement and assembly errors [37]. Cross-domain graph anomaly detection further explores graph transfer and graph decoupling to address anomalies across graph domains [38]. These developments broaden anomaly modeling across tasks and data types. For complex textured industrial products, integrating visual representations with reconstruction and normal-distribution modeling remains an important direction for exploiting complementary anomaly cues.

### 2.5. Summary and Research Gap

Although existing unsupervised anomaly detection methods have achieved remarkable progress, they generally characterize anomalies from a single perspective, such as reconstruction inconsistency, feature distribution deviation or semantic representation discrepancy. Such single-dimensional modeling is insufficient for resin cut-off wheel inspection, where defects simultaneously exhibit pixel-level texture variations, feature-level structural differences and distribution-level manufacturing fluctuations. Consequently, the following research gaps remain:

1. Existing resin cut-off wheel inspection methods still rely heavily on supervised learning or handcrafted features, limiting their applicability in real manufacturing environments with scarce defect samples.
2. Current unsupervised methods generally employ a single anomaly score and therefore cannot effectively characterize multi-level defects ranging from fine-grained texture anomalies to large-scale structural damage and batch-level distribution shifts.

To bridge these gaps, this paper proposes a three-dimensional anomaly scoring principle and develops the MemCLR-GANomaly framework, which jointly models pixel-, feature- and distribution-level anomaly information through adaptive multi-cue fusion.

## 3. Method

Existing single-scalar anomaly detection methods (e.g., GANomaly) compress heterogeneous anomaly cues into one scalar, leading to information aliasing. For resin cut-off wheel inspection, this design fails to simultaneously cover micro texture defects, meso structural damage and macro batch fluctuations, resulting in inherent detection blind spots. Targeted at this problem, we build the MemCLR-GANomaly framework based on the three-dimensional anomaly scoring principle. The network architecture and each scoring branch are detailed as follows.

### 3.1. Three-Dimensional Anomaly Scoring Principle

To address the information aliasing problem of single-scalar scoring mechanisms in multi-type resin cut-off wheel defect detection, this work proposes a three-dimensional anomaly scoring principle as the theoretical foundation. We posit that industrial anomalies manifest as joint deviations across three approximately complementary dimensions: pixel-level structure $D_{p}$ for local geometric distortions, feature-level semantics $D_{f}$ for appearance inconsistencies, and distribution-level statistics $D_{d}$ for global domain shifts.

The anomaly evidence is conceptually decomposed into three complementary dimensions:

(1) $$D\left(x\right)=\left[D_{p}\left(x\right),D_{f}\left(x\right),D_{d}\left(x\right)\right].$$

For cut-off wheel inspection, the three dimensions respectively match micro texture anomalies, structural defects and batch quality fluctuations, covering all typical defect categories across representation levels. Guided by this principle, Equation (1) is instantiated via three complementary scoring branches detailed in the following subsections.

### 3.2. Design of the MemCLR-GANomaly Framework

**Figure 1:**
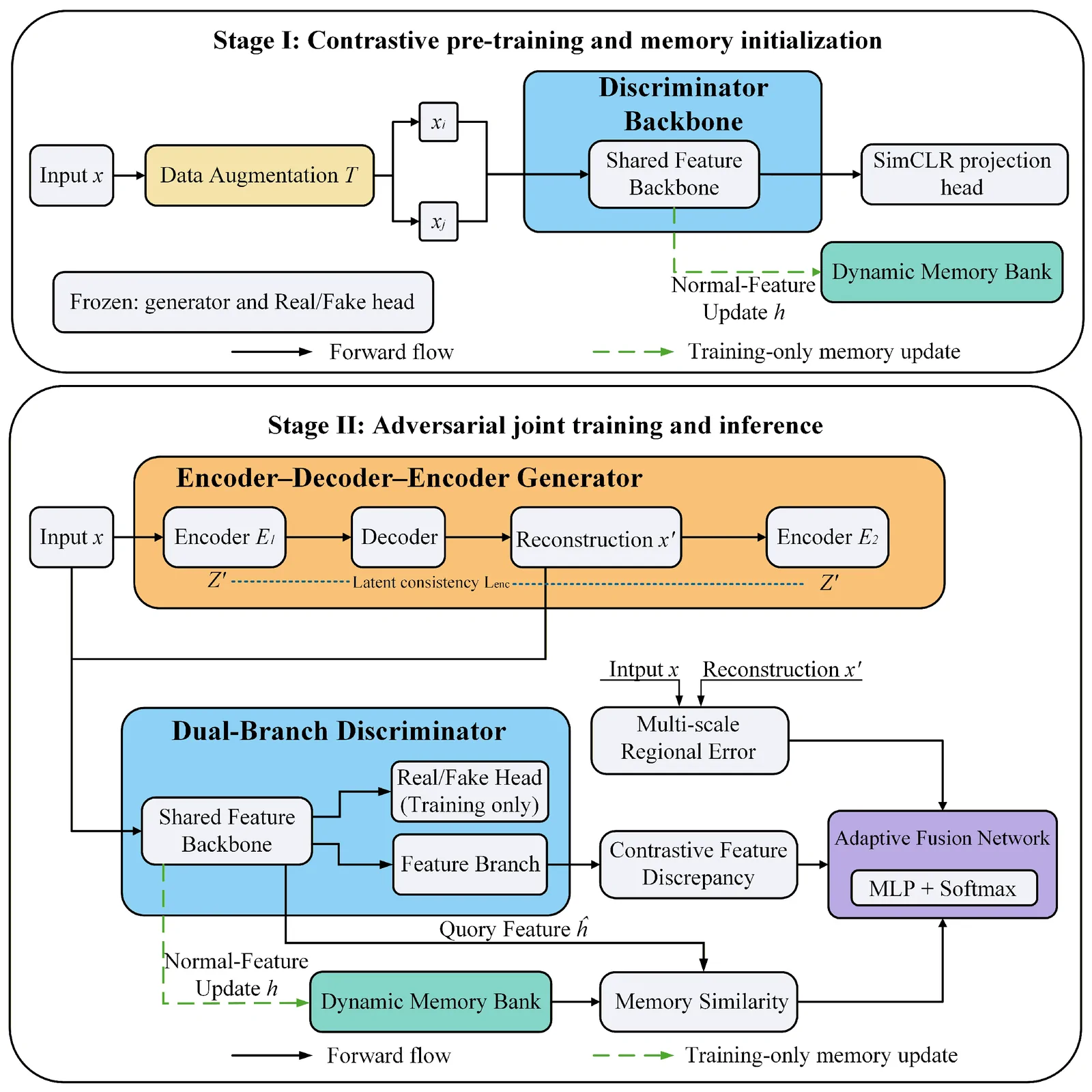
Overall framework and two-stage workflow of MemCLR-GANomaly.

As shown in Figure 1, MemCLR-GANomaly adopts a two-stage unsupervised anomaly detection framework. Stage I performs contrastive pre-training and memory initialization using augmented normal samples. In this stage, the discriminator backbone is optimized with a SimCLR projection head, and the extracted normal features are used to initialize the dynamic memory bank. Stage II covers adversarial joint training and inference. The input image is first reconstructed by an encoder–decoder–encoder generator. The original image, reconstructed image, discriminator features, and memory bank are then used to derive three complementary anomaly cues: multi-scale regional reconstruction error, contrastive feature discrepancy, and memory similarity.

These cues are integrated by an adaptive fusion network to obtain the final image-level anomaly score. By combining pixel-level reconstruction, feature-level representation comparison, and distribution-level normal-reference modeling, the framework reduces the limitations of single-score anomaly detection for multi-type resin cut-off wheel defects.

#### 3.2.1. Dual-Branch Discriminator Architecture with Contrastive Learning

In the original GANomaly, the discriminator mainly performs real/fake discrimination, which limits its ability to learn intrinsic normal representations. For resin cut-off wheels, complex mesh and printed label textures may obscure subtle defects. To enhance feature discriminability, the discriminator backbone is first pre-trained using SimCLR contrastive learning and then shared by the adversarial and feature-measurement branches.

As shown in Figure 1, the real/fake head outputs the probability $P_{\mathrm{real}}\in \left[0,1\right]$, preserving adversarial discrimination. The shared backbone produces the base representation h, which is used for contrastive feature discrepancy calculation and memory bank storage. During pre-training, a lightweight SimCLR projection head is attached to the shared backbone for contrastive loss optimization and is not used during inference.

This design retains adversarial supervision while introducing contrastive representation learning, providing discriminative embeddings for feature-level semantic deviation measurement and memory-based normal-reference modeling.

#### 3.2.2. Dynamic Memory Bank

**Figure 2:**
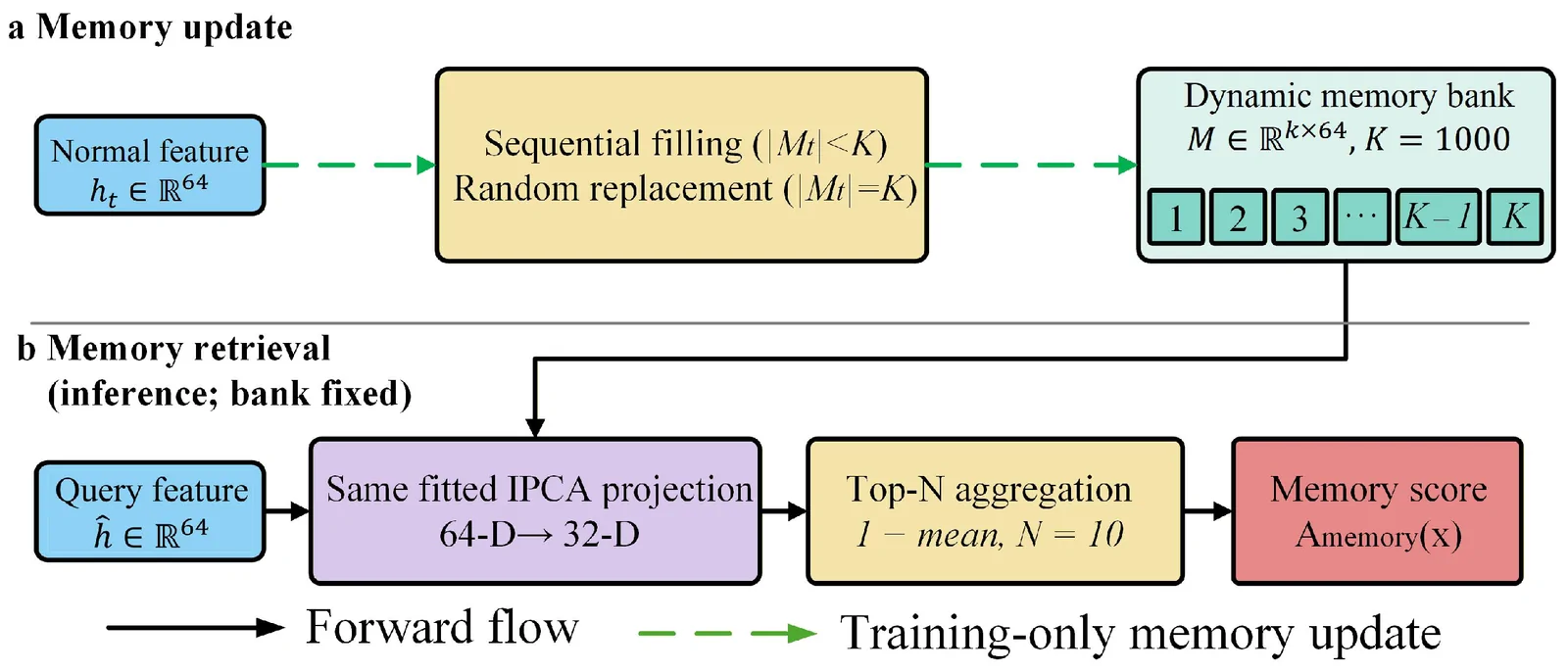
Update and retrieval mechanism of the dynamic memory bank.

To instantiate the distribution-level statistical dimension $D_{d}$ in Equation (1), we integrate a dynamic memory bank into the SimCLR framework. In resin cut-off wheel production, normal batch variations in resin matrix color and mesh compactness may cause false alarms when a fixed feature reference is used. The memory bank dynamically stores normal-sample feature vectors h extracted by the shared discriminator backbone. These features provide an empirical reference distribution of normal samples during inference. As illustrated in Figure 2, the mechanism comprises memory update during training and memory retrieval during inference.

The memory bank is represented as an updatable feature matrix $M\in R^{K\times d}$, where K=1000 is the maximum memory capacity and d=64 is the dimension of each stored feature vector. It is initialized as an empty set and updated using a sequential-fill-then-random-replacement strategy to maintain the diversity and timeliness of the stored normal prototypes.

Let $h_{t}$ denote the normal feature vector extracted at training iteration *t*. The memory bank is updated as

(2) $$M^{t+1}=\left\{\begin{array}{cc} \left[M^{t};h_{t}\right],\; & |M^{t}|<K,\\ \mathrm{Replace}\left(M^{t},r,h_{t}\right),\; & |M^{t}|=K, \end{array}\right.$$

where r∼U{1,…,K} is an index uniformly sampled from the memory slots. The operator Replace(·) replaces the *r*-th entry of $M^{t}$ with the incoming feature $h_{t}$. New features are appended until the memory reaches its maximum capacity. Once the memory is full, a randomly selected entry is replaced by the incoming feature.

##### IPCA-Based Dimensionality Reduction

To reduce the computational cost of memory retrieval, Incremental Principal Component Analysis (IPCA) is used to compress the stored 64D features to 32D:

(3) $$\tilde{M}=P_{\mathrm{IPCA}}\left(M\right),\;\tilde{M}\in R^{K\times 32},$$

where $P_{\mathrm{IPCA}}\left(\cdot \right)$ denotes the IPCA transformation fitted once on the normal training features extracted by the stabilized backbone after joint training. During inference, the memory bank remains fixed, and both the stored memory features and query feature are projected using the same fitted transformation.

### 3.3. Multidimensional Anomaly Score Computation

In conventional GANomaly, the anomaly score is derived solely from the latent vector difference, which cannot match the multi-level defect characteristics of resin cut-off wheel in industrial inspection. We therefore define anomaly evaluation along three complementary dimensions, each targeting a typical defect type in actual production: pixel-level error for surface texture defects, feature-level discrepancy for structural abnormalities, and distribution-level deviation for batch quality fluctuations. During inference, each test sample is evaluated via three pathways: (i) multi-scale region error, (ii) contrastive feature discrepancy, and (iii) memory bank similarity. An adaptive fusion network then integrates these scores into the final anomaly score A(x), adapting to different defect types on resin cut-off wheel end faces.

#### 3.3.1. Multi-Scale Region Error

To instantiate the pixel-level dimension $D_{p}$ in Equation (1), we design a multi-scale region error module. Pixel-level anomalies directly manifest as grayscale differences between input images and their reconstructions, and multi-scale partitioning is adopted to adapt to the wide size range of resin cut-off wheel defects, from sub-millimeter abrasive contamination to centimeter-scale glass fiber mesh damage.

We compute reconstruction errors across four patch scales: 2×2, 4×4, 8×8, and 16×16. Given an input image x and its reconstruction $x^{\prime }$, the mean absolute error (MAE, L1 norm) of the *p*-th region at a specific scale is calculated as:

(4) $$e_{\mathrm{region}}\left(p\right)=\frac{1}{hw}\sum_{i=1}^{h}\sum_{j=1}^{w}{∥x_{i,j}-x_{i,j}^{\prime }∥}_{1}$$

where *h* and *w* are the height and width of the current region, respectively, $x_{i,j}$ denotes the pixel value at position (i,j), and $e_{\mathrm{region}}\left(p\right)$ is the MAE of the *p*-th region. For each scale, we take the maximum MAE across all regions as the scale-level error $e_{s}$. We then aggregate errors across scales and take the global maximum as the multi-scale region anomaly score:

(5) $$A_{\mathrm{region}}\left(x\right)=\max\left(e_{2},e_{4},e_{8},e_{16}\right)$$

where $e_{s}$ denotes the maximum region error at scale *s*. This maximum-based aggregation preserves sensitivity to localized defects across different scales, although isolated reconstruction errors caused by normal texture variations may occasionally produce high regional responses.

#### 3.3.2. Contrastive Feature Discrepancy

To instantiate the feature-level semantic dimension $D_{f}$ in Equation (1), we define the feature discrepancy between the real image and its reconstruction using discriminative backbone features learned by SimCLR pre-training. For resin cut-off wheel defects such as mesh misalignment and label printing defects, the pixel-level difference is subtle but the structural semantic difference is significant. Feature-level measurement can identify such abnormalities more sensitively than pixel-level error alone.

The SimCLR projection head is retained during training but is not used during inference. All inference-time features are extracted from the shared discriminator backbone. Let x be a test image and $x^{\prime }$ its reconstruction. Both are fed into the shared discriminator backbone to obtain L2-normalized backbone feature vectors $h_{i}$ and $h_{j}$. The contrastive anomaly score is defined using cosine distance:

(6) $$A_{\mathrm{contrast}}\left(x\right)=1-\cos\left(h_{i},h_{j}\right)=1-\frac{h_{i}\cdot h_{j}}{{∥h_{i}∥}_{2}\cdot {∥h_{j}∥}_{2}}$$

#### 3.3.3. Memory Bank Similarity

The distribution-level anomaly score is obtained by comparing a test feature with the normal-feature distribution stored in the dynamic memory bank. For a test image x, the shared discriminator backbone extracts a query feature $\hat{h}\in R^{64}$. The same IPCA transformation used for the memory bank is then applied to obtain

(7) $$\tilde{h}=P_{\mathrm{IPCA}}\left(\hat{h}\right),\;\tilde{h}\in R^{32}.$$

The cosine similarity between the compressed query feature $\tilde{h}$ and the *i*-th compressed memory feature $\tilde{M}\left[i\right]$ is calculated as

(8) $$s_{i}=\frac{{\tilde{h}}^{T}\tilde{M}\left[i\right]}{{∥\tilde{h}∥}_{2}{∥\tilde{M}\left[i\right]∥}_{2}}.$$

The *N* highest similarities are selected and averaged to obtain the normal-reference score:

(9) $$s_{\mathrm{mem}}=\frac{1}{N}\sum_{i=1}^{N}s_{\left(i\right)},\;N=10,$$

where $s_{\left(i\right)}$ denotes the *i*-th highest similarity. The memory-based anomaly score is then defined as

(10) $$A_{\mathrm{memory}}\left(x\right)=1-s_{\mathrm{mem}}.$$

A larger value of $A_{\mathrm{memory}}\left(x\right)$ indicates a greater deviation from the stored normal-feature distribution. This distribution-level score complements the pixel- and feature-level anomaly cues used in the adaptive fusion framework.

#### 3.3.4. Adaptive Fusion Network

Since the three anomaly dimensions provide complementary evidence and may have different numerical scales, we introduce a lightweight adaptive fusion network to combine their responses. The reference statistics are computed exclusively from the normal training set. Specifically, the score of each branch is calibrated as

(11) $${\tilde{A}}_{k}\left(x\right)=\frac{A_{k}\left(x\right)}{\mu_{k}+\epsilon },\;k\in \left\{\mathrm{region},\mathrm{contrast},\mathrm{memory}\right\}$$

where $\mu_{k}$ is the mean score of the *k*-th branch over the normal training set, and $\epsilon =10^{-8}$ avoids numerical instability. The statistics $\mu_{k}$ are fixed after estimation and remain unchanged during inference. This calibration reduces first-order scale differences among the three branches without using validation or test samples.

The calibrated score vector is fed into a lightweight multilayer perceptron. Its output logits $z=\{z_{1},z_{2},z_{3}\}$ are converted into fusion weights by

(12) $$w_{i}=\frac{\exp(z_{i})}{\sum_{j=1}^{3}\exp\left(z_{j}\right)},\;i\in \left\{1,2,3\right\}$$

The final anomaly score is

(13) $$A\left(x\right)=w_{1}{\tilde{A}}_{\mathrm{region}}\left(x\right)+w_{2}{\tilde{A}}_{\mathrm{contrast}}\left(x\right)+w_{3}{\tilde{A}}_{\mathrm{memory}}\left(x\right)$$

Thus, A(x) is a constrained convex combination of the calibrated pixel-level, feature-level, and distribution-level anomaly evidence.

#### 3.3.5. Pixel-Level Anomaly Localization

For pixel-level anomaly localization, the spatial anomaly response is generated by integrating reconstruction-based structural differences and feature-level semantic deviations. The reconstruction branch provides a pixel-wise residual map by comparing the input image with its reconstructed output:

(14) $$M_{\mathrm{rec}}\left(u,v\right)={∥x\left(u,v\right)-\hat{x}\left(u,v\right)∥}_{2}.$$

where x(u,v) and $\hat{x}\left(u,v\right)$ denote the input and reconstructed pixel values at spatial position (u,v), respectively. The reconstruction residual captures local appearance and structural differences between the two images.

Intermediate convolutional feature maps from the shared discriminator backbone provide an additional spatial discrepancy measure. Let $F_{q}$ denote the feature map extracted from the input image, and let $F_{\mathrm{rec}}$ denote the feature map extracted from its reconstruction. The reconstructed feature map serves as the reference for location-wise comparison. Unlike global embeddings used for image-level scoring, these feature maps retain spatial information. The feature discrepancy is defined as

(15) $$M_{\mathrm{feat}}\left(u,v\right)=1-\frac{F_{q}{\left(u,v\right)}^{T}F_{\mathrm{rec}}\left(u,v\right)}{{∥F_{q}\left(u,v\right)∥}_{2}{∥F_{\mathrm{rec}}\left(u,v\right)∥}_{2}+\epsilon }$$

Here, $F_{q}\left(u,v\right)$ and $F_{\mathrm{rec}}\left(u,v\right)$ are the feature vectors of the input image and its reconstruction at spatial position (u,v), and ϵ ensures numerical stability.

The reconstruction and feature discrepancy maps are combined to form the initial pixel-level anomaly map:

(16) $$M_{\mathrm{pixel}}=M_{\mathrm{rec}}+M_{\mathrm{feat}}.$$

The normalized memory anomaly score provides image-level context for scaling the spatial response:

(17) $$M_{\mathrm{final}}=M_{\mathrm{pixel}}\left(1+\lambda A_{\mathrm{memory}}\right),$$

where λ controls the contribution of the memory score. A larger $A_{\mathrm{memory}}$ increases the overall response magnitude, while the reconstruction and feature discrepancy terms determine its spatial pattern. Adaptive fusion is used separately to compute the image-level anomaly score.

### 3.4. Training and Loss Function

#### 3.4.1. Overall Training Procedure

Our method follows a two-stage training strategy comprising contrastive pre-training and adversarial joint training.

Stage I: Contrastive Pre-Training

The generator and discriminator real/fake head are frozen, while the shared backbone and SimCLR projection head are optimized using InfoNCE on augmented pairs of normal images. Normal backbone features are simultaneously stored in the memory bank using the sequential-fill-then-random-replacement strategy.

Stage II: Adversarial Joint Training

The generator and discriminator are alternately optimized using their respective objectives. The memory bank is updated with normal features after each training batch. The fusion network is not optimized in this stage; it is trained afterward once the calibration statistics are fixed.

Algorithm 1 summarizes the training and inference procedures.
**Algorithm 1** Training and inference of MemCLR-GANomaly**Require:** 
Normal training data $X_{n}$ and test image x**Ensure:** 
Image-level anomaly score and pixel-level anomaly map   1:Initialize networks and an empty memory bank MStage I: Contrastive pre-training   2:Freeze the generator and discriminator real/fake head   3:**for** each normal mini-batch over the pre-training epochs **do**   4:    Generate paired augmentations and update the backbone and projection head using InfoNCE   5:    Append detached backbone features to M; when full, replace a random slot   6:**end for**Stage II: Adversarial joint training   7:Unfreeze the generator and discriminator real/fake head   8:**for** each normal mini-batch over the joint-training epochs **do**   9:    Alternately update the discriminator and generator using $L_{D}$ and $L_{G}$ 10:    Update M with detached normal backbone features 11:**end for**Post-training calibration and fusion training 12:Fit IPCA on normal features extracted by the final backbone; compress M to 32-D 13:Estimate calibration statistics $\mu_{k}$ on $X_{n}$ and freeze them 14:Train the fusion network on calibrated branch scores from $X_{n}$ using $L_{\mathrm{fusion}}$Inference 15:Freeze networks, memory bank, fitted IPCA, calibration statistics, and fusion network 16:Reconstruct x and extract the required features 17:Compute $A_{\mathrm{region}}$ and $A_{\mathrm{contrast}}$ 18:Project query and memory features using the same fitted IPCA 19:Compute $A_{\mathrm{memory}}$ using Top-*N* cosine similarities 20:Calibrate the three scores and compute the adaptive weighted sum 21:Construct the pixel-level anomaly map using the localization equations 22:**return** Image-level anomaly score and pixel-level anomaly map

### 3.4.2. Loss Functions and Optimization Objectives

#### Generator Objective

The total loss function of the generator consists of four weighted terms:

(18) $$L_{G}=w_{\mathrm{adv}}L_{\mathrm{adv}}+w_{\mathrm{enc}}L_{\mathrm{enc}}+w_{\mathrm{con}}L_{\mathrm{con}}+w_{\mathrm{contrast}}L_{\mathrm{contrast}}$$

where $w_{\mathrm{adv}}$, $w_{\mathrm{enc}}$, and $w_{\mathrm{con}}$ are fixed weighting coefficients, while $w_{\mathrm{contrast}}$ is a shared dynamic weighting coefficient used by both the generator and discriminator. It is updated during training as

(19) $$w_{\mathrm{contrast}}=\max\left(0.5,\;1-0.01\times \mathrm{epoch}\right),$$

This schedule gradually reduces the influence of the contrastive objective during joint training while maintaining a minimum contribution of 0.5.

(1) Feature matching loss.This loss constrains the generator to produce reconstructed images whose deep semantic features are consistent with those of real images at the discriminator’s final convolutional layer, promoting feature-level alignment between input and reconstruction:
  (20) $$L_{\mathrm{adv}}={∥f\left(x\right)-f\left(x^{\prime }\right)∥}_{2}$$
  where f(·) denotes the feature output of the last convolutional layer of the discriminator, *x* is the original input image, and $x^{\prime }$ is the corresponding reconstructed image.
(2) Pixel-level reconstruction loss. The L1 norm is adopted to measure the pixel-wise difference between the input image and its reconstructed counterpart, ensuring basic visual reconstruction fidelity:
  (21) $$L_{\mathrm{con}}={∥x-x^{\prime }∥}_{1}$$
(3) Latent encoding consistency loss. This loss enforces the latent vector of the reconstructed image to align with that of the original input, maintaining the stability and semantic consistency of the latent feature space:
  (22) $$L_{\mathrm{enc}}={∥z-z^{\prime }∥}_{2}^{2}$$
  where *z* is the latent vector of the input image encoded by the generator, and $z^{\prime }$ is the latent vector re-encoded from the reconstructed image.
(4) Contrastive InfoNCE loss. Derived from the SimCLR contrastive learning framework, this loss is applied to the augmented view pairs of the same image. It pulls positive sample features closer and pushes negative sample features apart, enhancing the discriminability of the feature space:
  (23) $$L_{\mathrm{contrast}}=-\log\frac{\exp\left(\mathrm{sim}(z_{i},z_{j})/\tau \right)}{\sum_{k=1}^{N}\exp\left(\mathrm{sim}(z_{i},z_{k})/\tau \right)}$$
  where $z_{i}$ and $z_{j}$ are the feature representations of two augmented views from the same image, *N* is the batch size, sim(·,·) denotes the cosine similarity function, and τ is the temperature parameter.

#### Discriminator Objective

The discriminator loss consists of a real/fake classification loss, a feature discrepancy loss, and a contrastive feature consistency loss:

(24) $$L_{D}=L_{\mathrm{cls}}+L_{\mathrm{feat}}+w_{\mathrm{contrast}}L_{\mathrm{match}}$$

where $w_{\mathrm{contrast}}$ shares the same definition as in Equation (19).

The real/fake classification loss is defined as

(25) $$L_{\mathrm{cls}}=-y\log D\left(x\right)-\left(1-y\right)\log\left(1-D\left(x^{\prime }\right)\right)$$

where D(x) and $D(x^{\prime })$ denote the probabilities that the original and reconstructed images are classified as real, respectively.

(1) Feature discrepancy loss ($l_{2}$ loss). This loss measures the $l_{2}$ distance between the deep features of real and reconstructed images. Note that the discriminator is optimized to maximize this loss to distinguish real samples from reconstructed ones, while the generator is optimized to minimize it:
  (26) $$L_{\mathrm{feat}}=-{∥f\left(x\right)-f\left(x^{\prime }\right)∥}_{2}$$
  where f(·) shares the same definition as in the generator’s feature matching loss.
(2) Contrastive feature MSE loss. This loss constrains the SimCLR branch of the discriminator to generate consistent features for different augmented views of the same image, maintaining the stability of contrastive feature representations:
  (27) $$L_{\mathrm{match}}={∥z_{i}-z_{j}∥}_{2}^{2}$$
  where $z_{i}$ and $z_{j}$ are the SimCLR branch features corresponding to two augmented views of the same input image.

#### Adaptive Fusion Network Objective

Following adversarial training in Stage II, the generator, discriminator, and memory bank are frozen. IPCA is fitted, and the calibration statistics are estimated from the normal training set. The fusion network is then optimized using the three calibrated branch scores. These scores are detached from the computation graph, so fusion optimization updates only the fusion network.

For a mini-batch of *B* normal samples, the fusion objective combines normal-response compactness and branch utilization balance:

(28) $$L_{\mathrm{fusion}}=L_{\mathrm{compact}}+\lambda_{\mathrm{bal}}L_{\mathrm{bal}},$$

where

(29) $$L_{\mathrm{compact}}=\frac{1}{B}\sum_{b=1}^{B}A\left(x_{b}\right)$$

minimizes the fused anomaly response on normal training samples. The balance term regularizes the average weight assigned to each branch:

(30) $$L_{\mathrm{bal}}=\sum_{i=1}^{3}{\left({\bar{w}}_{i}-\frac{1}{3}\right)}^{2},\;{\bar{w}}_{i}=\frac{1}{B}\sum_{b=1}^{B}w_{b,i},$$

where $w_{b,i}$ denotes the fusion weight of branch *i* for sample *b*. The coefficient $\lambda_{\mathrm{bal}}$ is fixed at 0.1 for both datasets.

The balance term regularizes the batch-averaged branch weights, while Softmax normalization ensures that the weights for each sample sum to one. At inference, the three branch scores are calibrated using the fixed normal-training statistics and combined using the weights predicted by the trained fusion network.

## 4. Experiments

### 4.1. Experimental Datasets

To comprehensively verify the effectiveness and generalization of the proposed method, experiments are conducted on two datasets: a custom resin cut-off wheel surface defect dataset and the public MVTec AD benchmark dataset. The custom dataset contains multiple surface defect categories collected under industrial conditions. All defect labels and pixel-level annotations were generated through manual annotation according to the observed defect regions.

#### 4.1.1. Resin Cut-Off Wheel Dataset

**Figure 3:**
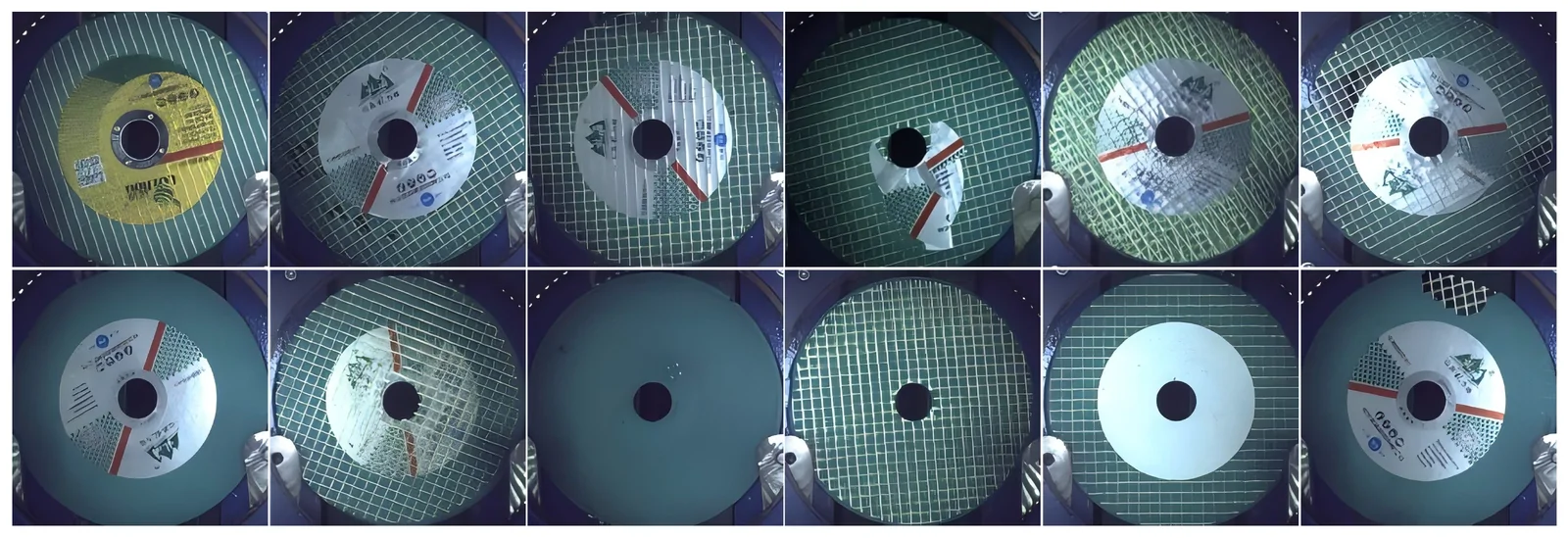
Typical samples of the self-built Resin Cut-off Wheel Dataset, covering label, mesh and structural anomaly types.

The Resin Cut-off Wheel Dataset contains 1700 images of resin-bonded reinforced cut-off wheels with a resolution of 750×750 pixels, including 600 normal and 1100 anomalous samples. The anomalous samples cover 11 anomaly types grouped into label, mesh, and structural anomalies. Representative samples are shown in Figure 3.

To reduce data leakage, images acquired consecutively within the same acquisition batch under unchanged production and imaging conditions are treated as one collection group. Groups are assigned as intact units according to acquisition order, ensuring that no group spans different subsets. The fixed partition contains 360 normal training images, 90 normal and 110 anomalous validation images, and 150 normal and 990 anomalous test images. The validation set is used only for post-training threshold selection and is not involved in model or hyperparameter optimization.

#### 4.1.2. MVTec AD Dataset

**Figure 4:**
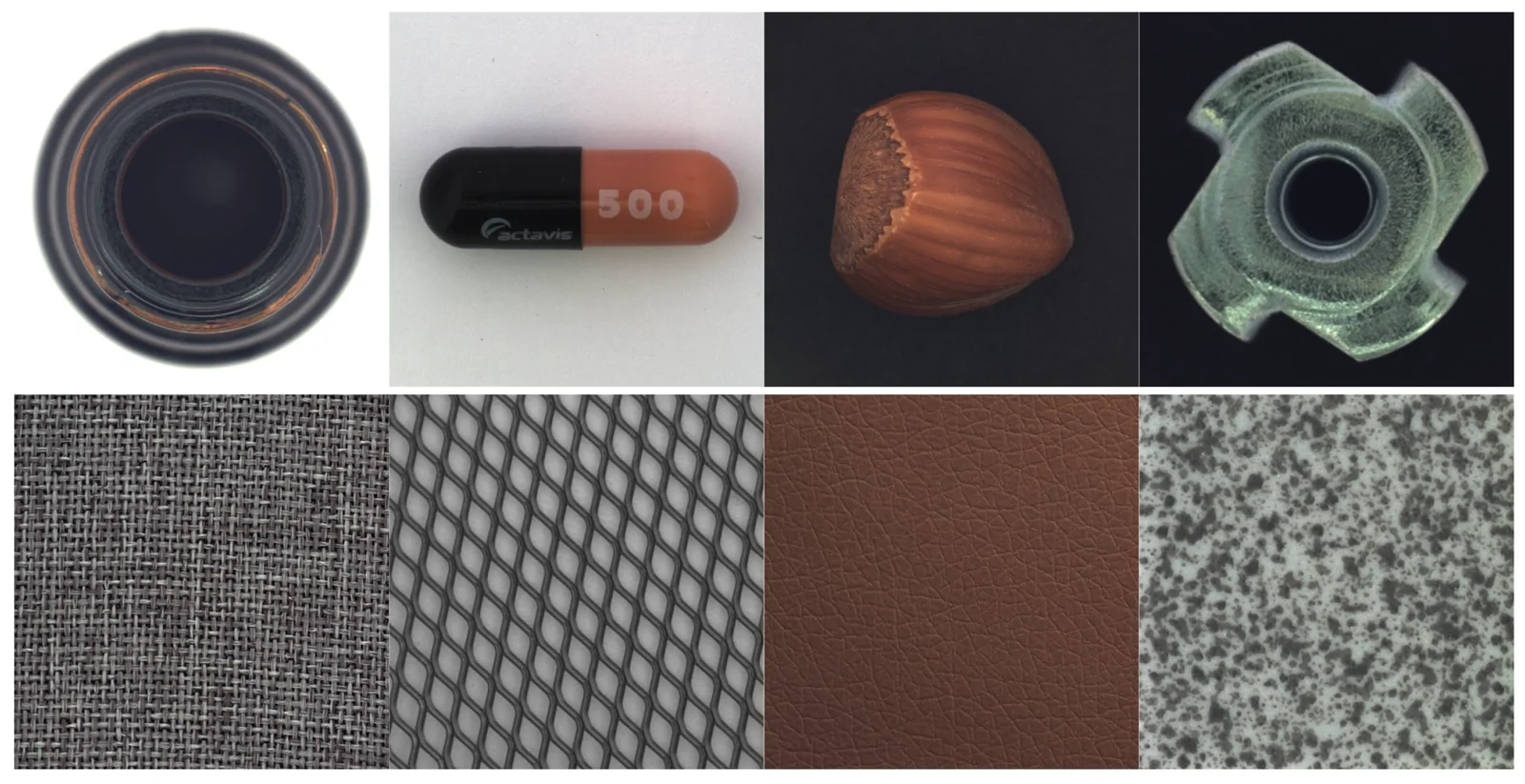
Representative samples of the MVTec AD dataset. The first row shows object categories (bottle, capsule, hazelnut, metal_nut), and the second row shows texture categories (carpet, grid, leather, tile).

The MVTec AD dataset is a widely used benchmark for unsupervised visual anomaly detection and localization, containing 15 categories. For each category, normal images from the official training and test subsets are pooled and repartitioned into training, validation, and test subsets at ratios of 70%, 10%, and 20%. Thus, normal images from the official test subset are included in the repartition. Anomalous images from the official test subset are divided into validation and test subsets at ratios of 10% and 90%. A separate model is trained for each category using only its normal training subset. All category-specific models use the same hyperparameter settings specified in Section 4.2.2. Anomaly thresholds are selected on each category’s validation set and fixed for evaluation on its held-out test set. Figure 4 shows representative samples from MVTec AD.

### 4.2. Implementation Details

#### 4.2.1. Data Preparation

All images are resized to 256×256 and normalized to [−1,1], with no test-time augmentation. Standard SimCLR augmentations (color jitter, grayscale, horizontal flip, Gaussian blur) are used only in Stage I pre-training; only random horizontal flip is applied in Stage II joint training.

#### 4.2.2. Parameter Configuration

The Adam optimizer with $\left(\beta_{1},\beta_{2}\right)=\left(0.5,0.999\right)$ is used with an initial learning rate of $1\times 10^{-4}$ and a batch size of 8. The model is first pre-trained for 50 epochs using contrastive learning, followed by 70 epochs of adversarial joint training. The temperature parameter in the InfoNCE loss is set to τ=0.1, the memory-bank capacity is K=1000, the IPCA output dimension is 32, the Top-*N* retrieval parameter is N=10, the pixel calibration coefficient is λ=1.0, and the branch-balance coefficient is $\lambda_{\mathrm{bal}}=0.1$. The fixed generator loss weights are set to $w_{\mathrm{adv}}=1$, $w_{\mathrm{enc}}=1$, and $w_{\mathrm{con}}=50$, following the standard GANomaly configuration. The nonstandard settings were selected based on normal-training behavior and computational considerations and were fixed before threshold selection; anomalous validation samples were not used in their selection. These settings are applied unchanged to MVTec AD. For each MVTec category, a separate set of model parameters is learned using only the corresponding normal training samples, without category-specific hyperparameter tuning.

#### 4.2.3. Reproducibility and Evaluation Protocol

All experiments conducted in this study are repeated five times with different random seeds for network initialization and data shuffling. Metrics are reported as five-run means, with standard deviations where indicated. For the Resin Cut-off Wheel Dataset, 95% confidence intervals are estimated using 1000 image-level bootstrap resamples. The same resampled image indices are applied to all five runs, and each metric is calculated per run and then averaged. The 2.5th and 97.5th percentiles define the interval. Normal and anomalous images are resampled separately for image-level AUROC. All pixels within each sampled image are retained for pixel-level AUROC, while FPR resampling uses only normal images. Welch’s two-tailed *t*-test (α=0.01) is used only for between-method comparisons with five run-level AUROC values obtained under the same data partition and evaluation protocol. It is not applied to literature-sourced MVTec AD baseline results.

#### 4.2.4. Software and Hardware Environment

Experiments run on an Intel i7-12700 CPU, NVIDIA RTX A5000 (24 GB VRAM), 32 GB RAM, with Python 3.8, PyTorch 1.8.2 and CUDA 11.1.4. Under the custom MVTec AD partition described in Section 4.1.2, the cumulative two-stage training time across all 15 category-specific models is approximately 5.6 h for one random seed, with a peak GPU memory usage of 5.8 GB.

### 4.3. Detection Performance on Resin Cut-Off Wheel Dataset

#### 4.3.1. Performance Analysis by Defect Category

Table 1 summarizes the mean image-level AUROC across three major defect categories on the Resin Cut-off Wheel Dataset. MemCLR-GANomaly achieves the highest AUROC in all categories, with an overall mean of 0.995 (95% image-bootstrap CI: [0.992, 0.998]). The overall image-level AUROC is significantly higher than that of each baseline (Welch’s two-tailed *t*-test, p<0.01).

Specifically, its performance advantage is most prominent on structural defects: compared with the best baseline PatchCore, the AUROC improvement remains evident, which verifies the stronger detection capability of the three-dimensional scoring framework for complex structural anomalies. The proposed method also maintains stable high performance on label defects and mesh defects, showing good adaptability to industrial defects with different visual characteristics.

#### 4.3.2. Overall Detection and Localization Performance

The proposed method achieves optimal performance across all six metrics. At the pixel level, it attains an AUROC of 0.988 (95% CI: [0.984, 0.992]), outperforming the strongest baseline PatchCore by 2.5 percentage points (see Table 2), demonstrating superior fine-grained localization capability for both small point defects and large-area structural anomalies. For image-level detection, the method reaches a macro-averaged precision of 0.984, recall of 0.979, and F1-score of 0.981 across 11 defect categories, maintaining an excellent balance between detection accuracy and completeness.

Notably, for industrial deployment-critical indicators, the proposed method yields a specificity of 0.988 and reduces the false positive rate (FPR) to 0.012 (95% CI: [0.006, 0.018]). Compared with PatchCore, the FPR is reduced by 1.9 percentage points, which may reduce manual re-inspection workload and associated operational costs in production. This advantage stems from the three-dimensional scoring framework, which cross-validates anomaly evidence across pixel, feature, and distribution subspaces, significantly suppressing background false alarms caused by normal texture variations.

**Figure 5:**
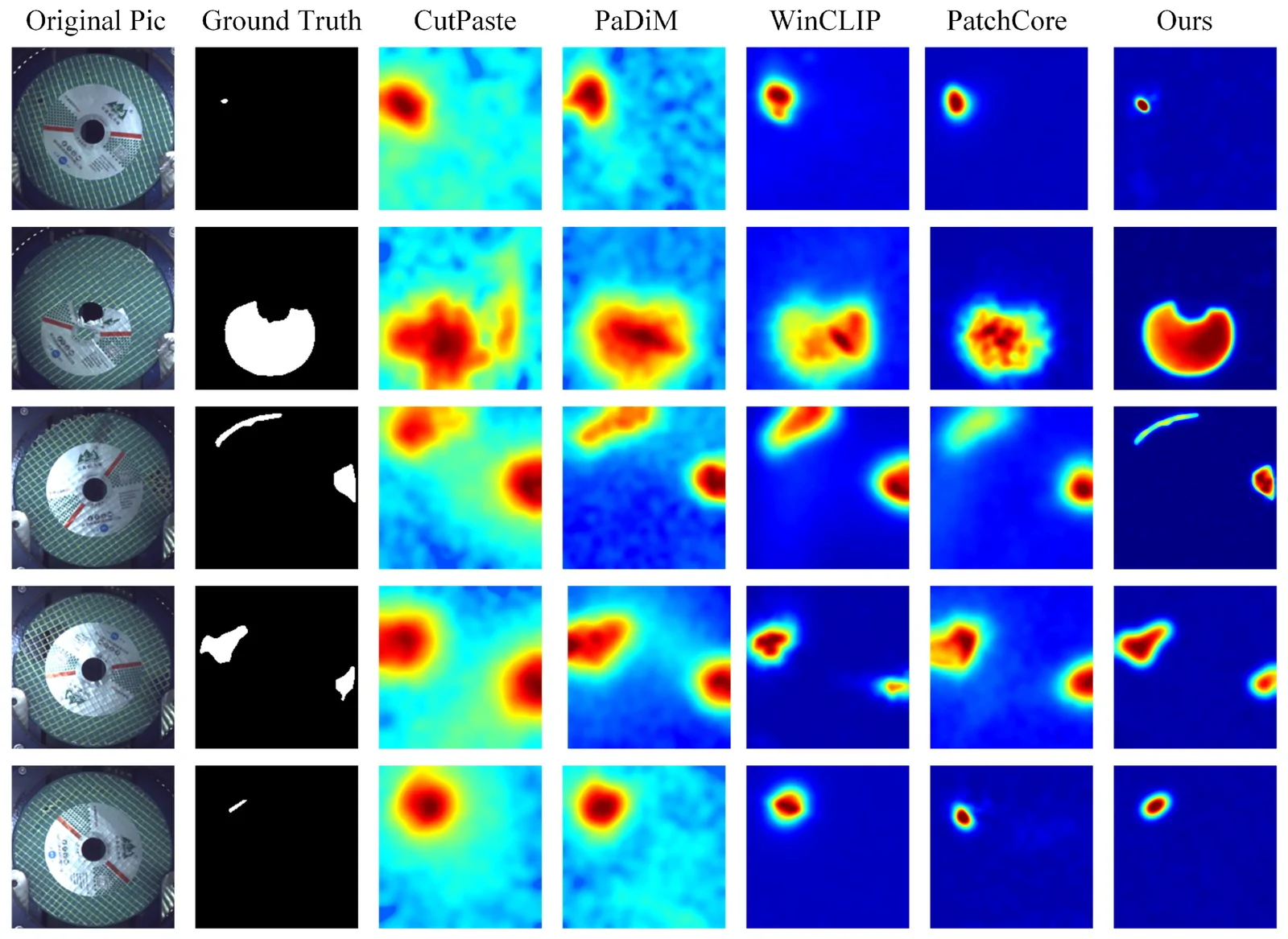
Visualization comparison of anomaly localization responses for different methods on the Resin Cut-off Wheel Dataset. In the heatmaps, blue indicates a low anomaly response and red indicates a high anomaly response; white regions in the ground-truth masks denote annotated defects.

Figure 5 visualizes anomaly localization results on five representative samples, covering label-area point defect, large-area label tear, arc-shaped mesh crack, irregular structural damage, and fine linear micro-crack. They span all three defect categories and cover pixel-level texture to feature-level structural anomalies. From left to right: input image, ground truth mask, and heatmaps from CutPaste, PaDiM, WinCLIP, PatchCore, and the proposed method.

Baseline methods show varying degrees of response diffusion and blurred boundaries. In contrast, our method produces heatmaps more consistent with ground truth, with sharper edges and more complete defect coverage. For the tiny point defect in the first row, baselines yield diffuse responses with heavy background noise, while our method achieves precise localization with minimal artifacts. For the large-area label tear in the second row, baselines present irregular blurred contours, whereas our method retains high shape consistency. For the arc-shaped mesh crack and irregular structural damage in rows 3 and 4, baselines suffer from over-diffusion and scattered false positives, while our method generates continuous, well-defined activation zones. For the fine linear micro-crack in the fifth row, baselines show weakened localization precision, while our method still preserves clear and complete defect boundaries.

The consistent superiority across these five visualized cases confirms that the three-dimensional scoring framework effectively integrates structural, semantic and distributional cues, delivering comprehensive performance gains for industrial inspection scenarios.

#### 4.3.3. Ablation Experiment

To assess the contribution of each core module in the proposed three-dimensional scoring framework, we conduct systematic ablation experiments on the Resin Cut-off Wheel Dataset. All variants are trained and evaluated under identical settings to ensure fair comparison.

Table 3 summarizes the empirical ablation results obtained under identical experimental settings. Among the three individual branches, the memory branch achieves the highest AUROC of 0.921. All pairwise combinations outperform their corresponding single branches, and the feature–memory pair achieves an AUROC of 0.956. The three-branch fixed-weight fusion achieves an AUROC of 0.975. Under the evaluated settings, the adaptive fusion configuration yields an AUROC of 0.995, an AP of 0.983, and an F1-score of 0.981.

#### 4.3.4. Visual Analysis of Memory Bank Update Strategy

**Figure 6:**
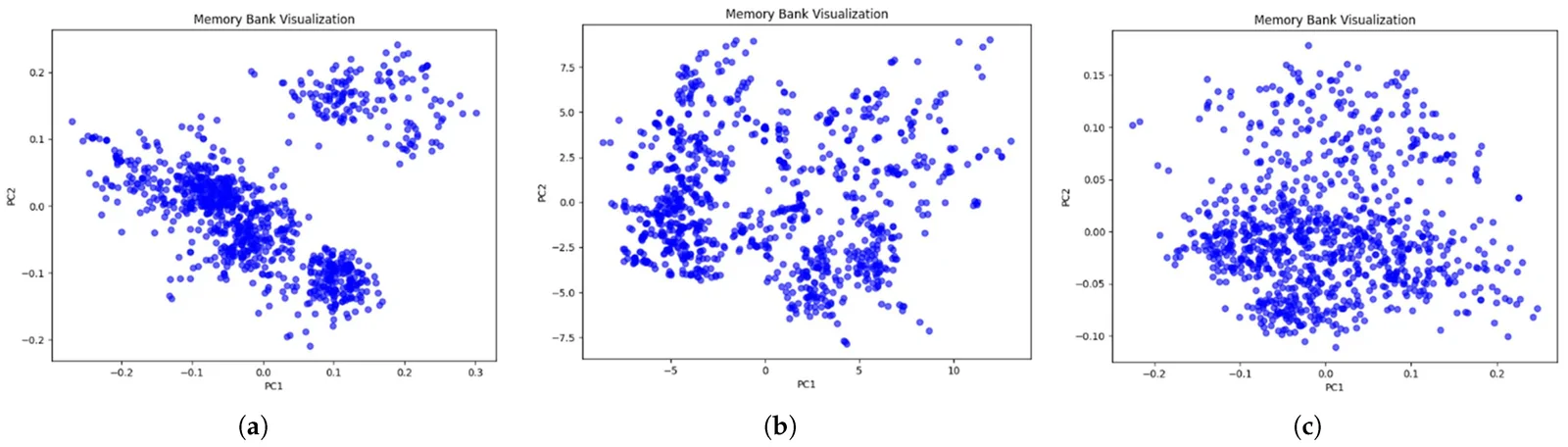
PCA visualization of memory bank features under different update strategies. (**a**) Pre-training update omitted. (**b**) Adversarial training update omitted. (**c**) Complete two-stage update strategy.

The superior performance of the memory branch stems from its dynamic two-stage update mechanism. To further interpret its internal working principle, we project the high-dimensional memory features into 2D space via PCA for comparison under different update strategies, as shown in Figure 6.

Under the complete two-stage update strategy (Figure 6c), the stored normal features are highly concentrated in the range PC1∈[−0.15,0.15] and PC2∈[−0.10,0.05], forming a compact distribution with high feature purity. When the pre-training update is omitted (Figure 6a), the feature distribution becomes more dispersed with obvious outlier clusters, covering approximately PC1∈[−0.2,0.2] and PC2∈[−0.15,0.15], indicating that the memory bank fails to establish a compact normal feature baseline at the initial stage. When the adversarial-training update is omitted (Figure 6b), features scatter widely across PC1∈[−5,10] and PC2∈[−7.5,7.5], which means the memory bank retains obsolete pre-training features and cannot adapt to the feature distribution evolution during adversarial training.

These visualization results validate that dynamic updates during adversarial training play a pivotal role in sustaining the timeliness and representativeness of memory prototypes. The full two-stage update scheme produces the most compact and generalizable normal feature distribution, which aligns well with the quantitative finding that the memory branch achieves the best single-branch performance.

#### 4.3.5. Interpretability of Adaptive Fusion Weights

**Figure 7:**
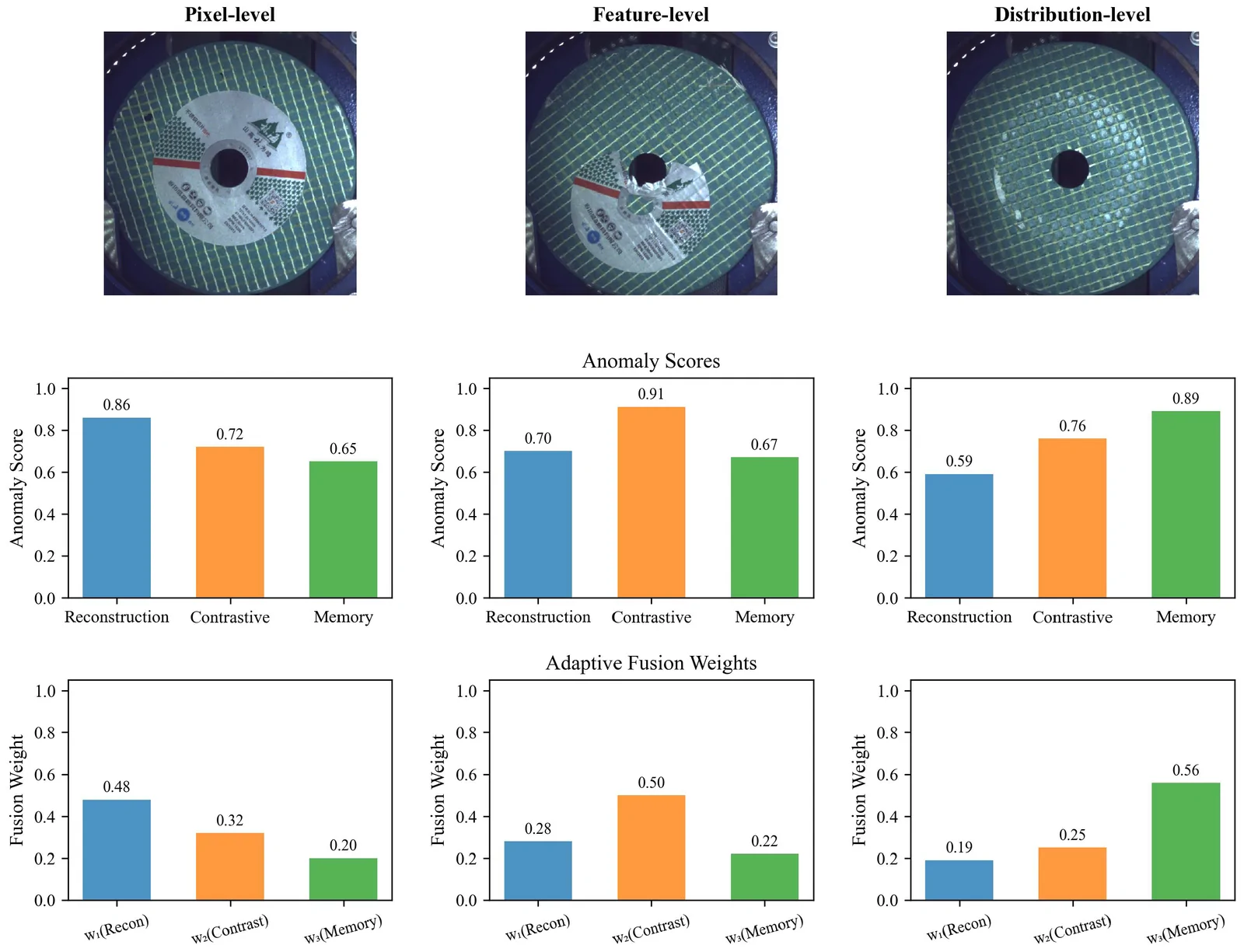
Complementary responses and adaptive fusion weights of three branches under different anomaly characteristics.

Beyond the complementarity of the three branches, the adaptive fusion network provides an additional mechanism for improving anomaly detection performance. To interpret its adaptive weighting behavior, Figure 7 visualizes the branch anomaly responses and the corresponding Softmax-normalized fusion weights for three representative anomaly types with different dominant dimensions.

For pixel-level texture defects, the reconstruction branch receives the largest contribution weight (0.48), indicating that local appearance variations are mainly captured by pixel-level reconstruction errors. For feature-level structural defects, the contrastive branch obtains the highest contribution weight (0.50), demonstrating the importance of high-level feature discrepancy modeling. For distribution-level batch shifts, the memory branch dominates with a contribution weight of 0.56, confirming the effectiveness of memory-based distribution representation.

The consistent correspondence between anomaly characteristics and the learned relative contributions demonstrates that the adaptive fusion network can dynamically adjust the importance of different anomaly dimensions according to the input characteristics. This adaptive weighting strategy further validates the complementarity of the three-dimensional anomaly scoring framework and explains its advantage over fixed-weight fusion approaches.

#### 4.3.6. Qualitative Localization Comparison

**Figure 8:**
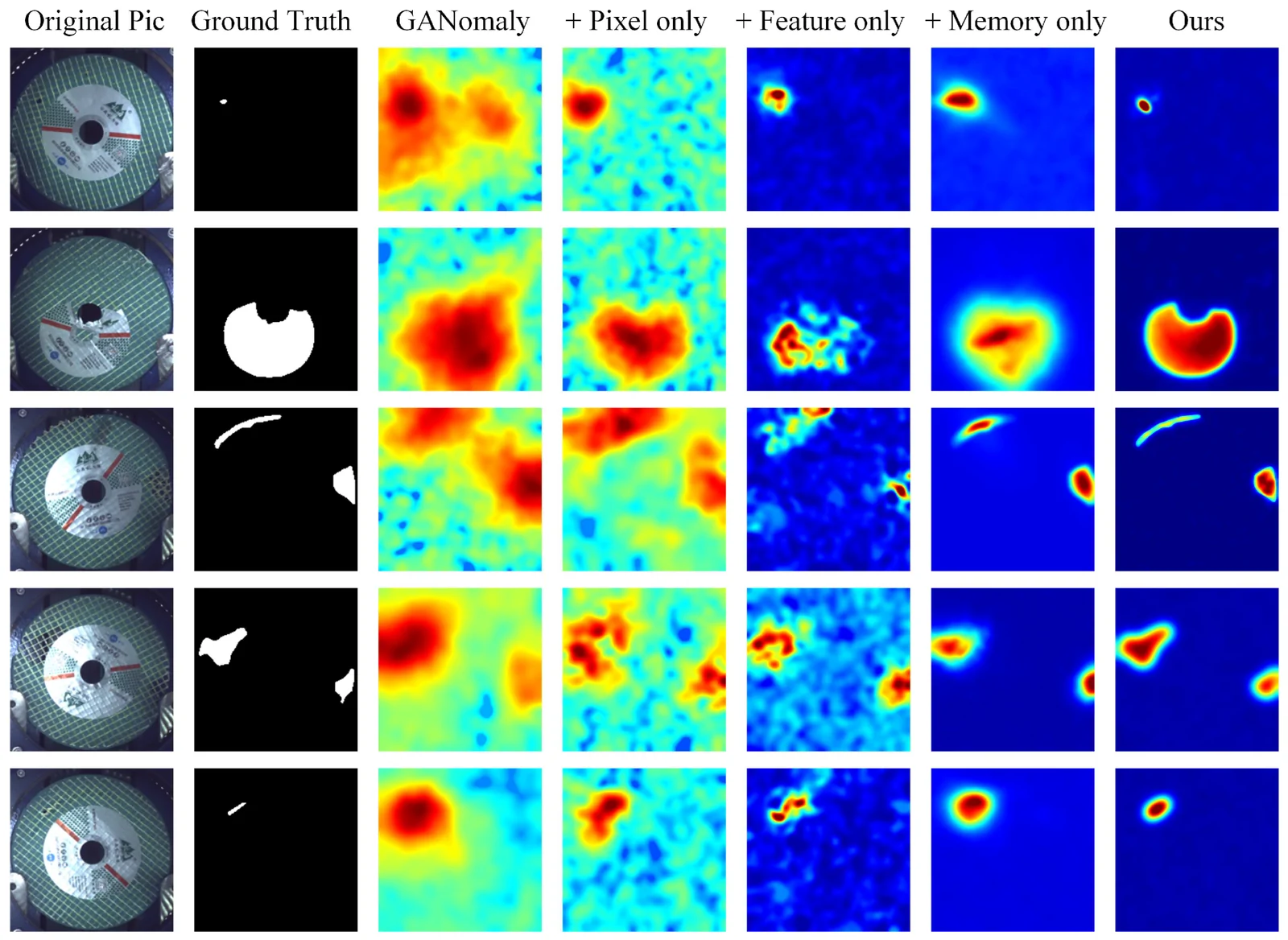
Qualitative localization comparison of the baseline, single-branch variants, and complete MemCLR-GANomaly model on representative defect samples. In the heatmaps, blue indicates a low anomaly response and red indicates a high anomaly response; white regions in the ground-truth masks denote annotated defects.

Figure 8 presents qualitative ablation results on five representative samples: a label-area point defect, a large-area label tear, an arc-shaped mesh crack with an accompanying structural defect, multi-region structural damage, and a fine linear micro-crack. Each row shows the input image, ground truth mask, and localization maps from the GANomaly baseline, single-branch variants, and complete model.

The GANomaly baseline exhibits diffuse responses and substantial background activation in the displayed examples. The pixel-branch variant responds to local structural differences but retains background interference, while the feature-branch variant shows more concentrated activation in several samples. The memory-augmented variant and complete model also exhibit differences in defect coverage and response concentration. In the selected examples, the complete model produces high responses near the annotated defects with relatively low background activation. These observations complement the quantitative results in Table 3, without establishing a uniform localization advantage across all defect types.

#### 4.3.7. Computational Efficiency Analysis

Table 4 compares the inference efficiency of different algorithms on our custom Resin Cut-off Wheel Defect Dataset. WinCLIP shows the lowest efficiency due to the heavy computational burden of its Transformer structure. Among feature-based baselines, PatchCore is slower than PaDiM and CutPaste owing to the nearest neighbor retrieval operation, and CutPaste achieves the best baseline speed of 40.2 FPS.

Benefiting from lightweight architecture design and optimized retrieval strategy, our method outperforms all baselines with 23.6 ms inference time, 42.3 FPS and 3.9 GFLOPs, fully meeting the real-time requirement of industrial online inspection.

#### 4.3.8. Robustness Verification

**Figure 9:**
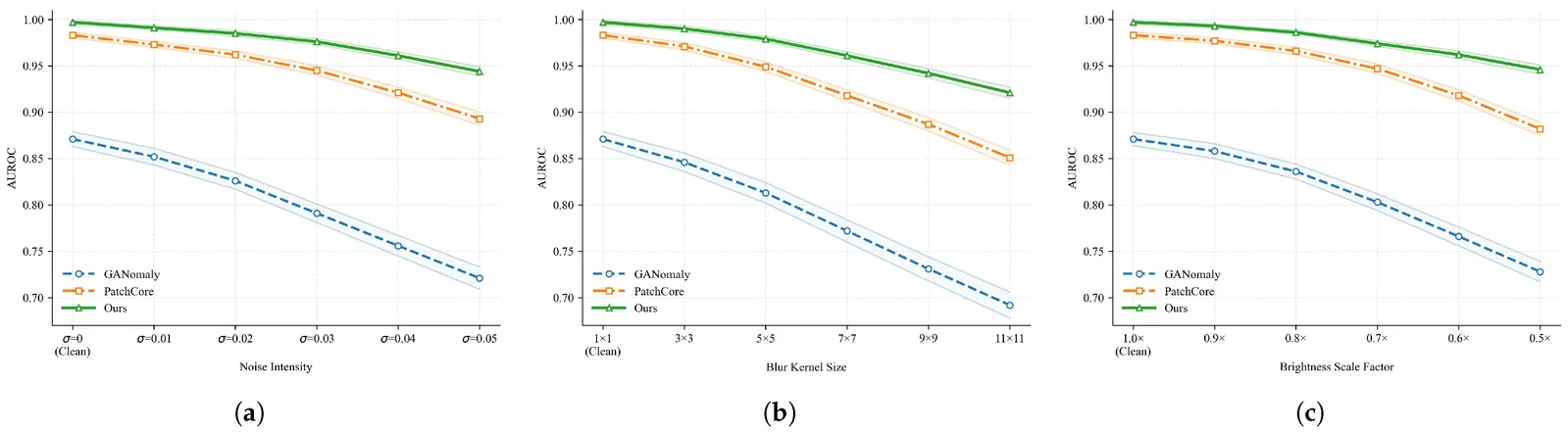
Robustness comparison of different methods under three types of image disturbances with increasing intensity. (**a**) Gaussian noise. (**b**) Gaussian blur. (**c**) Brightness shift.

To evaluate the environmental adaptability of the proposed method in industrial inspection scenarios, robustness tests were conducted under three common image disturbances: Gaussian noise, Gaussian blur, and brightness shift. Six intensity levels were used for each disturbance. For Gaussian noise, the standard deviation was set to σ∈{0,0.01,0.02,0.03,0.04,0.05}, where σ=0 denotes clean images. For Gaussian blur, the kernel size was set to k∈{1,3,5,7,9,11}, where 1×1 denotes the undegraded image. For brightness shift, the scale factor was set to λ∈{1.0,0.9,0.8,0.7,0.6,0.5}, where λ=1.0 denotes the original brightness. Figure 9 reports the mean AUROC at each disturbance level, with shaded regions indicating the standard deviation across repeated runs.

As shown in Figure 9, the AUROC of all methods decreases as the disturbance intensity increases, while the proposed method consistently maintains the highest mean AUROC. The relatively narrow error bands also indicate stable performance across repeated runs. Under Gaussian noise, GANomaly shows the largest degradation, whereas the proposed method preserves a smoother performance curve. Gaussian blur produces the strongest overall degradation, especially at large kernel sizes, but the proposed method remains clearly above the baselines. Under brightness variation, all methods are less affected than under blur, and the proposed method still retains the best robustness. These results indicate that combining reconstruction-, feature-, and memory-based cues improves tolerance to common imaging disturbances in industrial environments.

### 4.4. Anomaly Detection Performance on MVTec AD

MemCLR-GANomaly is compared with GANomaly, PaDiM, CutPaste, PatchCore, and WinCLIP on MVTec AD. Baselines follow their original papers or officially recommended configurations. The proposed method retains the hyperparameter configuration used for the Resin Cut-off Wheel dataset, with a separate model trained on normal samples from each MVTec category. Table 5 reports image-level (Det.) and pixel-level (Seg.) AUROC, with group and overall means calculated across the corresponding categories. Because these results are based on different data partitions and evaluation protocols, the table provides a contextual reference across the reported settings rather than a direct comparison under a common protocol.

Under the custom partition, MemCLR-GANomaly achieves overall image-level and pixel-level AUROC values of 0.948 and 0.936. Performance is higher on texture categories, with mean detection and localization AUROC values of 0.985 and 0.964, compared with 0.930 and 0.922 on object categories. Strong detection results are obtained for grid, leather, and tile, whereas cable, capsule, and pill remain challenging. These results support applicability beyond resin cut-off wheels while revealing category-dependent limitations.

**Figure 10:**
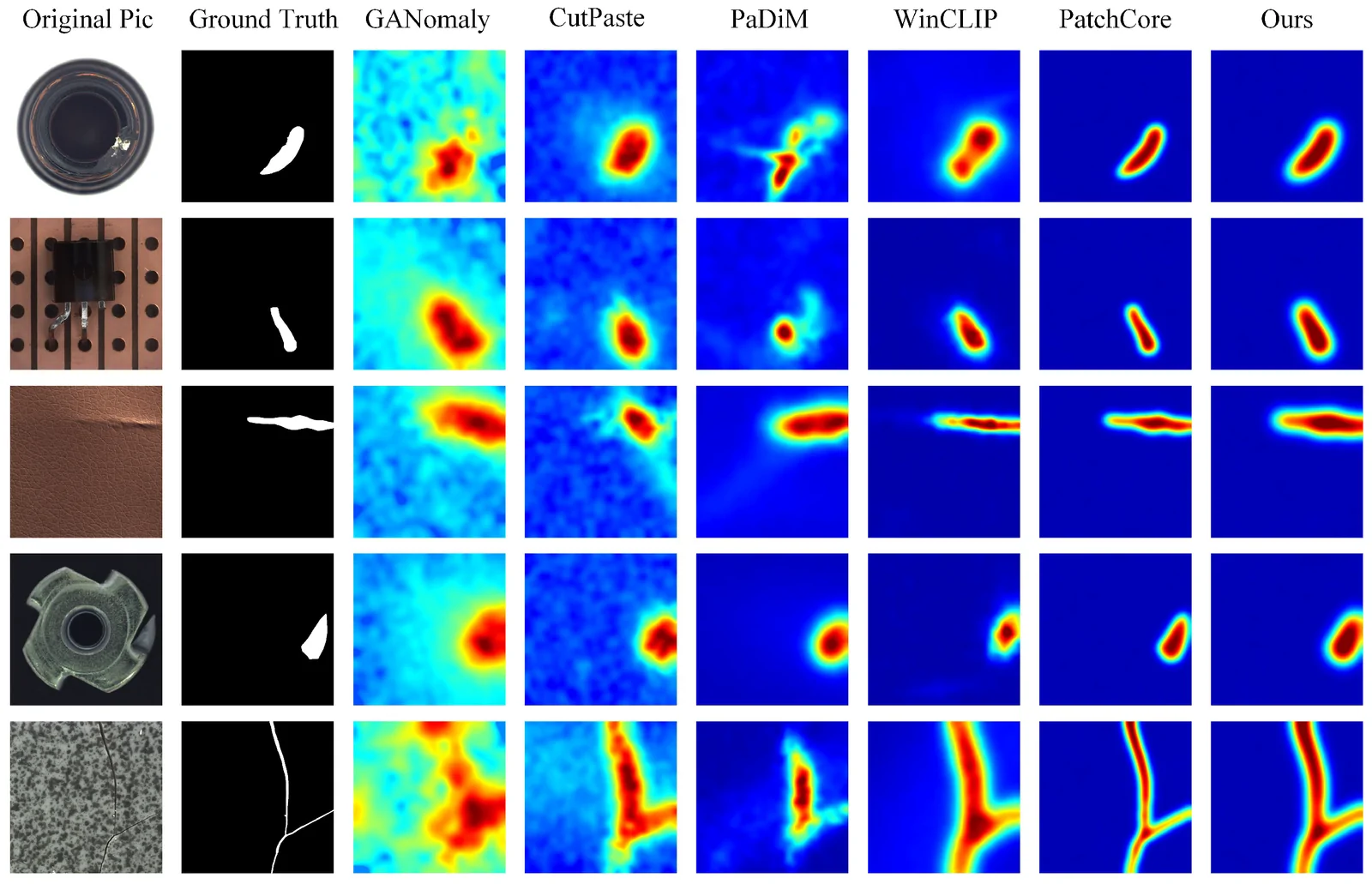
Visualization comparison of anomaly localization results on the MVTec AD dataset. In the heatmaps, blue indicates a low anomaly response and red indicates a high anomaly response; white regions in the ground-truth masks denote annotated defects.

Figure 10 presents localization results on object and texture samples from MVTec AD. GANomaly exhibits widespread background activation, reducing defect-background contrast. CutPaste and PaDiM identify anomalous regions but show dispersed responses or incomplete coverage, while WinCLIP captures the main defect locations with varying response extent. MemCLR-GANomaly highlights principal defect regions with relatively low background activation and captures both compact defects and elongated anomalies in textured samples. Compared with PatchCore, its responses are broader around defect boundaries in several examples, indicating room for improvement in boundary delineation.

## 5. Conclusions

This paper presents MemCLR-GANomaly for unsupervised surface inspection of resin cut-off wheels. The framework combines reconstruction error, contrastive feature discrepancy, and memory-based normality estimation through adaptive score fusion. On the self-built Resin Cut-off Wheel Dataset, the method achieves an image-level AUROC of 0.995 and a pixel-level AUROC of 0.988, outperforming the evaluated baselines. Under the custom MVTec AD partition, it achieves an image-level AUROC of 0.948 and a pixel-level AUROC of 0.936. Its mean image-level and pixel-level AUROC values are 0.985 and 0.964 for the texture categories and 0.930 and 0.922 for the object categories. The ablation results support the benefit of combining complementary anomaly cues. The measured inference speed of 42.3 FPS suggests potential for online inspection on the evaluated hardware.

Limitations: Performance varies across MVTec AD categories, with lower mean detection and localization performance on object categories than on texture categories. Broader responses around defect boundaries are observed in some examples. Severe Gaussian blur reduces detection performance, while robustness to long-term production drift and deployment on edge devices remain unverified.

Future Work: Future work will focus on refining spatial localization, improving robustness to imaging disturbances and production drift, and evaluating lightweight deployment on edge devices. Streamlining the training procedure will also be investigated.

## Figures and Tables

**Table 1:** Average AUROC results by defect category on the Resin Cut-off Wheel Dataset. Bold values indicate the best performance in each row.

Defect Category	GANomaly	CutPaste	PaDiM	WinCLIP	PatchCore	Ours
Label defects	0.871±0.008	0.916±0.005	0.967±0.004	0.954±0.006	0.986±0.002	0.996±0.002
Mesh defects	0.882±0.007	0.925±0.004	0.975±0.003	0.968±0.005	0.988±0.002	0.996±0.001
Structural defects	0.850±0.010	0.899±0.007	0.926±0.005	0.933±0.007	0.978±0.004	0.994±0.003
Overall mean	0.868±0.008	0.913±0.006	0.956±0.003	0.952±0.006	0.984±0.002	0.995±0.002

**Table 2:** Overall industrial inspection and localization performance. Precision, recall, and F1-score are macro-averaged over 11 defect categories; specificity and FPR are computed on the entire normal test set.

Method	Pixel AUROC ↑	Precision ↑	Recall ↑	F1-Score ↑	Specificity ↑	FPR ↓
GANomaly	0.843	0.792	0.756	0.774	0.873	0.127
CutPaste	0.881	0.847	0.822	0.835	0.901	0.099
PaDiM	0.924	0.912	0.892	0.903	0.945	0.055
WinCLIP	0.914	0.905	0.882	0.894	0.935	0.065
PatchCore	0.963	0.949	0.933	0.941	0.969	0.031
Ours	0.988	0.984	0.979	0.981	0.988	0.012

*Note:* An upward arrow indicates that a higher value is better, whereas a downward arrow indicates that a lower value is better. Bold values indicate the best performance in each column.

**Table 3:** Ablation study of the proposed anomaly scoring components.

Configuration	Pixel	Feature	Memory	Adaptive	AUROC	AP	F1-Score
Baseline (GANomaly)	—	—	—	—	0.868	0.792	0.774
+Pixel only	✔			Single	0.891	0.818	0.789
+Feature only		✔		Single	0.898	0.825	0.804
+Memory only			✔	Single	0.921	0.870	0.840
Pixel + Feature	✔	✔		Equal-weight	0.914	0.856	0.845
Pixel + Memory	✔		✔	Equal-weight	0.943	0.892	0.880
Feature + Memory		✔	✔	Equal-weight	0.956	0.921	0.894
Full (fixed weights)	✔	✔	✔	Equal-weight	0.975	0.952	0.937
Full (adaptive ours)	✔	✔	✔	Adaptive	**0.995**	**0.983**	**0.981**

*Note:* A check mark indicates that the corresponding component is included. Bold values indicate the best performance in each column.

**Table 4:** Comparison of inference efficiency on the Resin Cut-off Wheel Dataset.

Method	WinCLIP	PatchCore	PaDiM	CutPaste	Ours
Inference (ms) ↓	358.4	32.8	27.5	24.9	23.6
FPS ↑	2.8	30.5	36.4	40.2	42.3
FLOPs (G) ↓	16.9	6.1	5.2	4.5	3.9

*Note:* An upward arrow indicates that a higher value is better, whereas a downward arrow indicates that a lower value is better. Bold values indicate the best performance in each row.

**Table 5:** Image-level (Det.) and pixel-level (Seg.) AUROC on MVTec AD. Group and overall results are category-wise macro-averages.

Category	GANomaly		PaDiM		CutPaste		WinCLIP		PatchCore		Ours	
	Det.	Seg.	Det.	Seg.	Det.	Seg.	Det.	Seg.	Det.	Seg.	Det.	Seg.
*Texture categories*												
carpet	0.636	0.601	0.994	0.989	0.931	0.983	0.998	0.955	0.989	0.991	0.976	0.957
grid	0.834	0.752	0.957	0.949	0.999	0.975	0.995	0.925	0.975	0.975	0.991	0.981
leather	0.847	0.803	0.998	0.991	1.000	0.995	0.999	0.978	1.000	0.991	0.995	0.978
tile	0.721	0.648	0.974	0.912	0.934	0.905	0.999	0.965	0.994	0.949	0.994	0.974
wood	0.850	0.801	0.988	0.936	0.986	0.955	0.999	0.920	0.989	0.936	0.969	0.928
Texture mean	0.778	0.721	0.982	0.955	0.970	0.963	0.998	0.949	0.989	0.968	0.985	0.964
*Object categories*												
bottle	0.893	0.852	0.998	0.981	0.983	0.976	0.982	0.945	1.000	0.983	0.993	0.976
cable	0.638	0.551	0.922	0.958	0.806	0.900	0.889	0.850	0.993	0.989	0.848	0.855
capsule	0.751	0.703	0.915	0.983	0.962	0.974	0.723	0.800	0.976	0.985	0.863	0.875
hazelnut	0.640	0.602	0.933	0.977	0.973	0.973	0.975	0.900	1.000	0.984	0.954	0.932
metal nut	0.532	0.451	0.958	0.967	0.993	0.931	0.987	0.920	0.997	0.977	0.942	0.928
pill	0.659	0.602	0.944	0.947	0.924	0.957	0.912	0.850	0.959	0.977	0.865	0.870
screw	0.785	0.701	0.844	0.974	0.863	0.967	0.864	0.820	0.949	0.986	0.889	0.905
toothbrush	0.773	0.702	0.972	0.987	0.983	0.981	0.922	0.880	1.000	0.972	0.989	0.955
transistor	0.727	0.651	0.975	0.972	0.955	0.930	0.834	0.800	0.999	0.949	0.967	0.942
zipper	0.668	0.601	0.908	0.982	0.994	0.993	0.888	0.880	0.992	0.984	0.989	0.978
Object mean	0.707	0.642	0.937	0.973	0.944	0.958	0.898	0.865	0.987	0.979	0.930	0.922
**Overall mean**	0.730	0.668	0.952	0.967	0.952	0.960	0.931	0.893	0.987	0.975	0.948	0.936

## Data Availability

The custom Resin Cut-off Wheel Dataset is available from the corresponding author upon reasonable request. The MVTec AD dataset is publicly available.
